# Optical absorption in array of Ge/Al-shell nanoparticles in an Alumina matrix

**DOI:** 10.1038/s41598-019-56673-8

**Published:** 2020-01-09

**Authors:** Vito Despoja, Lovro Basioli, Jordi Sancho Parramon, Maja Mičetić

**Affiliations:** 1 Institute of Physics, Bijenička 46, HR-10000 Zagreb, Croatia; 2 Donostia International Physics Center (DIPC), P. Manuel de Lardizabal, 20018 San Sebastian, Basque Country Spain; 3 Department of Physics, University of Zagreb, Bijenička 32, HR-10000 Zagreb, Croatia; 4 Ruđer Bošković Institute, Bijenička cesta 54, HR-10000 Zagreb, Croatia

**Keywords:** Nanophotonics and plasmonics, Quantum dots, Nanoparticles

## Abstract

The absorption spectra in array of Ge, Al and Ge/Al-shell nanoparticles immersed in alumina (Al_2_O_3_) matrix is calculated in framework of *ab initio* macroscopic dielectric model. It is demonstrated that absorption is strongly enhanced when germanium nanospheres are encapsulated by Al-shell. Two absorption peaks, appearing in the spectra, correspond to low energy *ω*_+_ and high energy *ω*_−_ plasmons which lie in visible and ultraviolet frequency range, respectively. It is demonstrated that in Ge/Al-shell composite the *ω*_+_ plasmon exists only because quantum confinement effect which provides larger Ge band gap (Δ ~ 1.5 eV) and thus prevent decay of *ω*_+_ plasmon to continuum of interband electron-hole excitation in semiconducting core. Absorption in visible frequency range enhances additional 3 times when alumina is replaced by large dielectric constant insulator, such as SiC, and additional 6 times when Ge core is replaced by wide band-gap insulator, such as Si_3_N_4_. Strong enhancement of optical absorption in visible frequency range make this composites suitable for optoelectronic application, such as solar cells or light emitting devices. The simulated plasmon peaks are brought in connection with peaks appearing in ellipsometry measurements.

## Introduction

Metallic nanoparticles of subwavelength dimensions (considering the wavelength up to UV) due to their reduced dimensionality support dipolar collective electronic modes called localized surface plasmon resonances (LSPR) which can be excited by incident electromagnetic field. The LSPR produce strong evanescent electrical field which can enhance light absorption or light emission in adjacent semiconductor or chemical or biological environment. This is why LSPR in metallic nanoparticles in last two decades have been applying to enhance absorption in conventional or organic photovoltaics^1–3^ or to enhance light emission in light-emitting device^4,5^. Strong evanescent field in metallic nanoparticles can enhance the emission in weakly fluorescent biomolecules such that it also has been applying in biological sensing^6–9^. Metallic nanostructures can serve as sensing platform even for single molecule detection. For example, following the LSPR frequency shift caused by its interaction with molecular excitons can be exploited for accurate single-molecule detection^10,11^.

Semiconductor nanoparticles, on the other hand, are very applicable in numerous modern nanotechnology devices. They show confinement effects, so their properties can be easily tuned by their size. Especially interesting are Ge quantum dots, as they show very strong confinement effects. They are very applicable in high-efficient energy conversion devices and IR photodetectors^12,13^.

Nanoparticles with core/shell structure are especially interesting due to the new degree of freedom in creating their properties caused by interaction of core and shell. It has been shown recently that Ge/Si core/shell nanoparticles show strongly enhanced absorption with different position of absorption peaks, as well as longer exciton lifetime than in pure Ge or Si nanoparticles^14–16^. The reason is separation of excited electron and hole in core and shell of the nanoparticle, that is caused by its specific structure. These materials therefore are excellent candidate for application in high-efficient photovoltaic devices. A variety of these interesting properties and applications require extensive experimental and theoretical research of the optical properties of metallic nanostructures and especially of related nano-heterostructures which could have desired plasmonic or some other interesting absorption properties.

In this paper we present the theoretical simulation of optical absorption in lattice of Ge/Al-shell nanoparticles immersed in alumina (Al_2_O_3_) matrix. Each Ge/Al-shell nanoparticle consists of germanium spherical core encapsulated by aluminium spherical shell. We explore the absorption in nanoparticles of various size and shell thickness. For comparison, the absorption in lattice of Ge/Al-shell nanoparticles is compared with absorption in lattice of Ge and Al spherical nanoparticles and with absorption in array of Ge/Al-shell nanoparticles where Ge core and Al_2_O_3_ matrix are replaced by other semiconductors. It is shown that absorption is strongly enhanced when germanium nanospheres are encapsulated by Al-shell. In absorption spectra dominates two peaks which correspond to low energy *ω*_+_ and high energy *ω*_−_ plasmons which lie in visible and ultraviolet frequency range, respectively. The appearance of these peaks closely resembles the excitation of bonding and anti-bonding modes in metal-dielectric core-shell systems as result of hybridization of sphere and cavity like modes^17^ It is demonstrated that in Ge/Al-shell composite the *ω*_+_ plasmon exists only because quantum confinement effect which provides larger Ge band gap (Δ ~ 1.5 eV) and thus prevent decay of *ω*_+_ plasmon to continuum of interband electron-hole excitation in semiconducting core. Absorption in visible frequency range enhances additional 3 times when alumina is replaced by large dielectric constant insulator, such as SiC, and additional 6 times when Ge core is replaced by wide band-gap insulator, such as Si_3_N_4_. Strong enhancement of absorption in visible frequency range makes this composites suitable for optoelectronic application, e.g. in photovoltaic or light emitting devices. The simulated plasmon peaks are brought in connection with peaks appearing in ellipsometry measurements.

The theoretical predictions are well supported by experimental work. A series of thin films consisting of Ge/Al-shell nanoparticles embedded in alumina matrix were produced by magnetron sputtering deposition^18^. The films differ by Al-shell thickness and Ge/Al-shell nanoparticles are regularly ordered in 3D lattice. The optical absorption in Ge/Al-shell lattice significantly differ from absorption in pure Ge nanoparticles lattice in the same matrix, and also depends on the shell thickness, as predicted by theory.

The theoretical simulation is provided in the framework of *ab initio* macroscopic dielectric model. This means that dielectric response of each particular component in heterostructure is described by local, macroscopic dielectric function *ϵ*_*i*_(*ω*), calculated from first principles. The response of whole heterostructure (e.g. Ge-sphere/Al-shell/Al_2_O_3_-matrix) is described by effective dielectric function *ϵ*_*eff*_(*ω*) derived by solving the Poisson equation and implementing the spherical boundary conditions. The electromagnetic energy dissipation rate or optical absorption is derived from imaginary part of effective dielectric function, *P* ∝ ℑ*ϵ*_*eff*_(*ω*). In Sec. 2 we present the methodology used to calculate the macroscopic and effective dielectric functions (*ϵ*_*i*_ and *ϵ*_*eff*_) of array of Ge/Al-shell nanoparticles in Al_2_O_3_-matrix. In Sec. 3 we present the results for optical absorption in array of Al spheres, Ge spheres and Ge/Al-shell nanoparticles of various radii and shell thicknesses. The advantages of using other semiconductors such as SiC, SiO_2_ and Si_3_N_4_ instead of Ge and Al_2_O_3_ are studied. Finally, the simulated spectra are compared with experiment. In Sec. 4 we present our concluding remarks.

## Modeling of the System

In the first stage of the modeling we obtain the macroscopic dielectric functions *ϵ*_*i*_(*ω*) of bulk *i* = Ge, Al and Al_2_O_3_ crystals by using *ab initio* methodology. Then by solving Poisson equation for boundary conditions we determine the effective macroscopic dielectric function *ϵ*_*eff*_ of Ge-core/Al-shell/Al_2_O_3_-matrix composite.

### *Ab initio* calculation of bulk crystals dielectric response

To calculate the Kohn-Sham (KS) wave functions *ϕ*_*n*k_ and energy levels *E*_*n*_(k), i.e. the band structure, of bulk *i* = Ge, Al and Al_2_O_3_ crystals, we use the plane-wave self-consistent field DFT code (PWSCF) within the QUANTUM ESPRESSO (QE) package^19^. The core-electron interaction is approximated by the norm-conserving pseudopotentials^20^ and the exchange correlation (XC) potentials are approximated in Ge by hybrid BLYP^21^, in Al_2_O_3_ by Perdew-Burke-Ernzerhof generalized gradient approximations (GGA)^22^ and in Al by Perdew-Zunger local density approximation (LDA)^23^ functionals. The crystal structures used in the calculation are; for Ge diamond cubic FCC (with two Ge atoms in unit cell) with lattice constant *a* = 5.66 *Å*, for Al cubic FCC with lattice constant *a* = 4.05 *Å* and for aluminium-oxide (Al_2_O_3_) hexagonal (12 Al and 18 O atoms in unit cell) with lattice constants *a* = 4.76 *Å* and *c* = 12.99 *Å*. The ground state electronic densities of Ge and Al crystals are calculated using the 8 × 8 × 8 and of Al_2_O_3_ crystal using 9 × 9 × 3 Monkhorst-Pack^24^ K-point mesh sampling of the first Brillouin zone (BZ). For all crystals the plane-wave cut-off energy is chosen to be 50Ry (680 eV).

The 3D Fourier transform of independent electrons response function is given by

1 $${\chi }_{{\bf{G}}{\bf{G}}{\boldsymbol{^{\prime} }}}^{0}({\bf{q}},\omega )=\frac{2}{\Omega }\sum _{{\bf{k}}\in B\mathrm{}.Z\mathrm{}.}\sum _{n,m}\,\frac{{f}_{n}({\bf{k}})-{f}_{m}({\bf{k}}+{\bf{q}})}{\omega +i\eta +{E}_{n}({\bf{k}})-{E}_{m}({\bf{k}}+{\bf{q}})}\times {\rho }_{n{\bf{k}},m{\bf{k}}+{\rm{q}}}({\bf{G}})\,{\rho }_{n{\bf{k}},m{\bf{k}}+{\rm{q}}}^{\ast }({\bf{G}}{\boldsymbol{^{\prime} }}),$$

where *f*_*n***K**_ = ${[{e}^{({E}_{n{\bf{K}}}-{E}_{F}/kT}+1]}^{-1}$ is the Fermi-Dirac distributions at temperature *T*. The matrix elements are

2 $${\rho }_{n{\bf{k}},m{\bf{k}}+{\bf{q}}}({\bf{G}})={\langle {\varphi }_{n{\bf{k}}}|{e}^{-i({\bf{q}}+{\bf{G}}){\bf{r}}}|{\varphi }_{n{\bf{k}}+{\rm{q}}}\rangle }_{\Omega },$$

where **q** is the momentum transfer wave vector and integration is performed over the normalization volume Ω. Plane wave expansion of the wave function has the form ${\varphi }_{n{\bf{k}}}({\bf{r}})=\frac{1}{\sqrt{\Omega }}{e}^{i{\bf{kr}}}\,{\sum }_{G}{C}_{n{\bf{k}}}({\bf{G}}){e}^{i{\bf{Gr}}}$, where **G** are 3*D* reciprocal lattice vectors, *r* is a 3*D* position vector, and the coefficients *C*_*n***k**_ are obtained by solving the Kohn-Sham equations selfconsistently. From response matrix (1) we determine the dielectric matrix

3 $${ {\mathcal E} }_{{\bf{G}}{\bf{G}}{\boldsymbol{^{\prime} }}}({\bf{q}},\omega )={\delta }_{{\bf{G}}{\bf{G}}{\boldsymbol{^{\prime} }}}-\sum _{{{\bf{G}}}_{1}}\,{v}_{{\bf{G}}{{\bf{G}}}_{1}}({\bf{q}}){\chi }_{{{\bf{G}}}_{1}{\bf{G}}{\boldsymbol{^{\prime} }}}^{0}({\bf{q}},\omega ),$$

where bare Coulomb interaction is ${v}_{{\bf{G}}{\bf{G}}{\boldsymbol{^{\prime} }}}({\bf{q}})=\frac{4\pi }{|{\bf{q}}+{\bf{G}}{|}^{2}}\,{\delta }_{{\bf{G}}{\bf{G}}{\boldsymbol{^{\prime} }}}$. Finally the macroscopic dielectric function of particular crystal can be determined by inverting the dielectric matrix

4 $$\epsilon (\omega )={\epsilon }_{1}(\omega )+i{\epsilon }_{2}(\omega \mathrm{)=1/}{ {\mathcal E} }_{{\bf{g}}=0{\bf{G}}{\boldsymbol{^{\prime} }}\mathrm{=0}}^{-1}({\bf{q}}\approx \mathrm{0,}\omega \mathrm{)}.$$

The probability density *P*(**q**,*ω*) for the parallel momentum transfer **q** and the energy loss *ω* of the electron in Electron Energy Loss Spectroscopy (EELS) experiments^25^ is proportional to the imaginary part of the dynamically screened Coulomb potential $W=v/\epsilon$, usually called the Electron Energy Loss Function (EELF). In optical limit (interested here) the EELF can be expressed in term of macroscopic dielectric function

5 $$P({\bf{q}}\approx 0,\,\omega )\propto -\Im \frac{1}{\epsilon (\omega )}\mathrm{}.$$

The wave vector **k** summation in the response function (1) is performed by using 41 × 41 × 41 *k*-point mesh sampling for Ge and Al crystals. Band summations (*n*,*m*) are performed over 20 bands for Al and over 30 bands for Ge. For Al_2_O_3_ response function calculation we have used 21 × 21 × 7 *k*-point mesh sampling and band summations are performed over 120 bands. The damping parameter used in all calculation is *η* = 100 meV and temperature is *T* = 10 meV. For optically small wave vectors (q ≈ 0), used in this modeling, the crystal local field effects are negligible, so the crystal local field effects cutt-off energy is set to be zero.

### Macroscopic dielectric functions *ϵ*_*i*_

In order to facilitate understanding the particular features in various nanoparticle/alumina-matrix composites we shall first analyze the dielectric functions and EELF of particular bulk crystals. Since in the considered composites the germanium will appear in the form of several nanometers large sphere it will be subject of strong quantum-confinement^26,27^, i.e. Ge nanoparticles band gap should be larger than bulk Ge band-gap. In order to capture this effect the Ge band-gap is (in accordance with ref. ^26^) increased from Δ = 0.66 eV to Δ = 1.5 eV and in some cases up to Δ = 3.0 eV. The band gap is increased simply such that conduction and valence bands are lifted up and down for equal value, as proposed in ref. ^26^, but the band structure is left unchanged. The germanium dielectric function *ϵ*_*Ge*_(*ω*) is then calculated using the same procedure (1–4). Even the shift in band-gap Δ causes just the same horizontal shift in local dielectric function *ϵ*_*M*_, we shall see, it will not be the case in effective dielectric function *ϵ*_*eff*_ because of the screening effects included through the boundary conditions. For example, if in bulk Germanium local dielectric function *ϵ*_*Ge*_ the interband peak appears at *ω*_0_ it will not necessarily appear, due to boundary screening effects, at the same frequency in effective dielectric function *ϵ*_*eff*_. In this way we shall include both microscopic quantum effects but also macroscopic screening. This approximation we believe is sufficient to model the dielectric response of studied heterostructure. Other possibility would be performing the *ab initio* calculation of nonlocal dynamical susceptibility tensor $\hat{\chi }$(*r*, *r*′*ω*) for entire heterostructure, which (in lowest approximation) implies solving of Kohn-Sham equation for unit cell which consists of thousands of Si and Al atoms. This is still impossible to provide by using existing computer resources.

**Figure 1:**
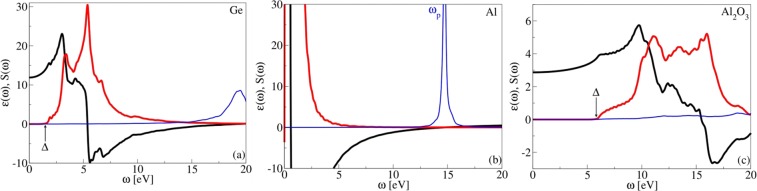
Real part ${\epsilon }_{1}(\omega )$ (black), imaginary part ${\epsilon }_{2}(\omega )$ (red) of macroscopic dielectric function and EELF (thin blue) of bulk crystals (**a**) Ge, (**b**) Al and (**c**) Al_2_O_3_. The germanium band gap is due to quantum-confinement increased to Δ = 1.5 eV.

Figure 1(a–c) show the Ge, Al and Al_2_O_3_ dielectric functions. One can notice characteristic insulator-like character, the static *ϵ*_1_(*ω* ≈ 0) has finite value and remains almost constant until band gap energy, which is Δ ≈ 1.5 eV for Ge and Δ ≈ 6 eV for Al_2_O_3_. The *ϵ*_2_ is zero until *ω* = Δ when *σ* → σ^*^ interband transitions starts to contribute and *ϵ*_2_ increases. The *ϵ*_2_ of Ge shows sharp peaks at $\omega  \sim 3.3$ and 5.3 eV which corresponds to transitions between Van Hove singularities in *σ* and σ^*^ bands. Because there is no intensive collective modes, such as plasmons, to which external field can be coupled the semiconducting EELF does not show any intensive peak. The Al dielectric function is shown in Fig. 1(b). One can notice characteristic metallic-like behavior, the static *ϵ*(*ω* ≈ 0) shows Drude singularity coming from infinite number of soft electron-hole intraband transitions within parabolic *s* or *σ* band. Also, due to lack of interband *s* → *d* transitions the Drude peak is only contribution in *ϵ*_2_. The Al EELF shows very strong plasmon peak at *ω*_*p*_ ≈ 15 eV (where *ϵ*_2_ also crosses zero), whose energy is consistent with simple jellium model predictions. For example, for aluminum, Wigner-Seitz radius is *r*_*s*_ = 2.07^28^ and the jellium plasmon frequency is *ω*_*p*_ = 3/*r*_*s*_^3^ = 15.8 eV which agrees well with the *ab initio* result.

### Modeling of the effective dielectric function *ϵ*_*eff*_

The dynamical electronic response and therefore optical properties of very small (*R*_*C*_ < 2 nm) nanoparticles are entirely defined by its microscopic electronic structure. Then the *ω* Fourier transform of local polarisation in such system caused by external electrical field **E**^*ext*^(**r**,*ω*) is

$${\bf{P}}(r,\omega )=\int d{{\rm{r}}}_{1}\hat{\chi }({\rm{r}},{{\rm{r}}}_{1},\omega )\otimes {{\bf{E}}}^{ext}({{\bf{r}}}_{1},\omega ),$$

where $\hat{\chi }$is nonlocal dynamical susceptibility tensor which depends on nanoparticle discrete set of energy levels and wave functions {*E*_*n*_, *ϕ*_*n*_(**r**)}. So the response and optical properties (e.g. optical absorption *P* ~ −*ωImχ*) strongly depends on nanoparticle shape and dimension because it defines its discrete electronic spectrum^29^. In this case the quantum confinement effect simulated just by horizontal shift of set of occuped and unoccuped energy levels would lead to incorrect optical spectra. On the other hand the dynamical response of sub-macroscopic (*R*_*C*_ > 10 nm) nanoparticles can be modeled by local polarisability

$${\bf{P}}({\rm{r}},\omega )=\chi ({\rm{r}},\omega ){{\bf{E}}}^{ext}({\rm{r}},\omega ),$$

which if we eliminate the crystal-local field effects or variation of dynamical response within unit cell can be additionally simplified and express in term of macroscopic dielectric function *χ*_*i*_(*ω*) = [${\epsilon }_{M}^{i}(\omega )$ − 1]/(4*π*) where indices *i* = 1, 2, 3, ... represent different region in nanopaticle heterostructure. The spatial distribution at nanoparticle boundaries is then included by applying boundary conditions. In this investigation the nanoparticles size is *R*_*C*_ ~ 2 nm such that we are faced with the problem of intermediate case, between mentioned two limits. However, we believe that the described local limit, in which microscopic effects are preserved through intra and interband excitations included in macroscopic dielectric functions ${\epsilon }_{M}^{i}(\omega )$, while quantum confinement will be phenomenological included through variable HOMO-LUMO band gap, is still valid.

**Figure 2:**
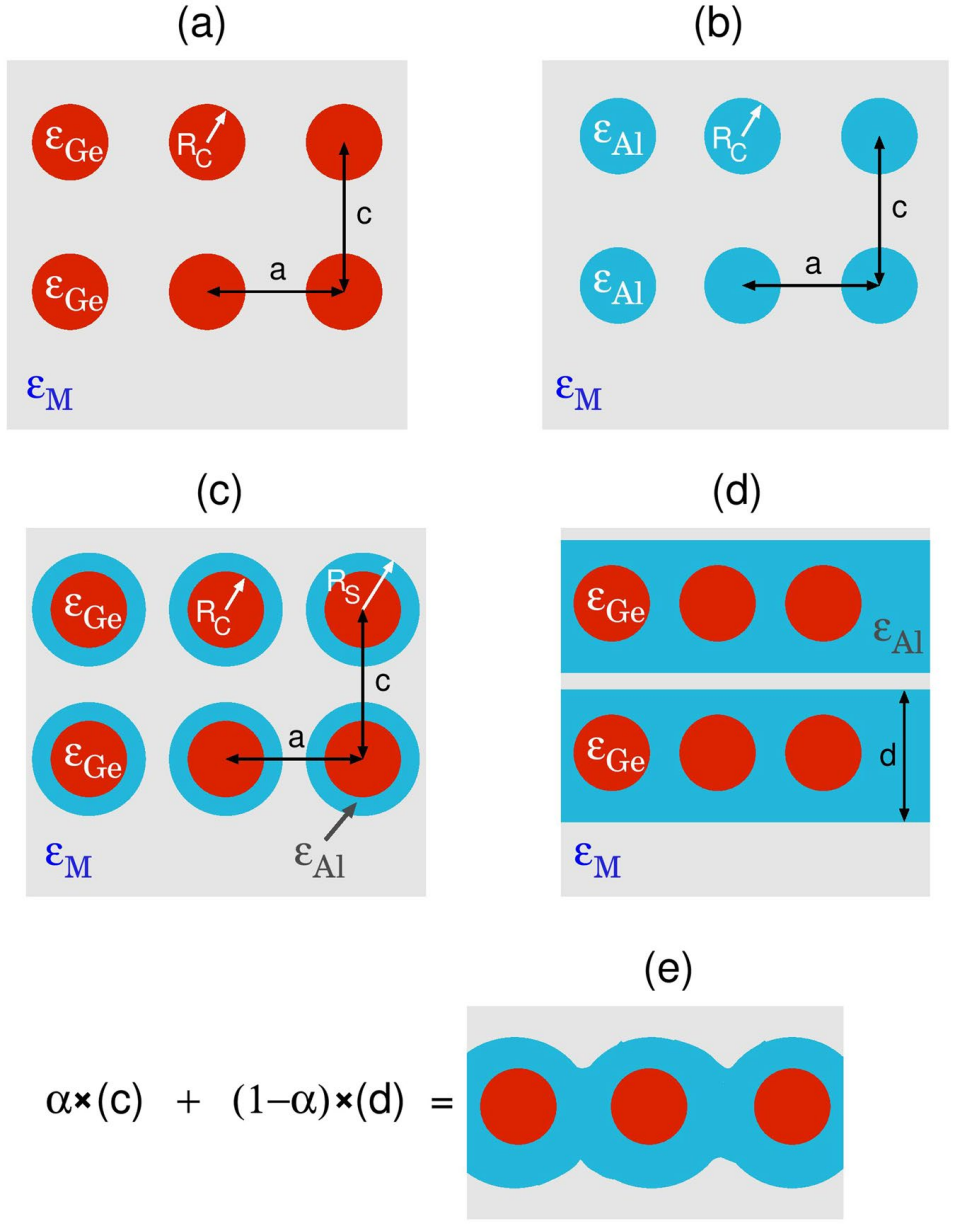
Modeling of the various Ge/Al nanoparticles placed in alumina (Al_2_O_3_) matrix. (**a**) Spherical Ge and (**b**) spherical Al nanoparticles of radius *R*_*C*_. (**c**) Spherical Ge-core/Al-shell nanoparticles of inner radius *R*_*C*_ and outer radius *R*_*S*_. (**d**) For larger Al concentrations the Ge/Al nanoparticles can be modeled as Ge spheres placed in metallic slab of thickness *d*. (**e**) Effective dielectric function of more realistic Ge/Al nanoparticles (for larger Al concentrations) can be modeled as linear combination of effective dielectric functions of model systems (**c**) and (**d**). The response properties of Ge and Al nanoparticles, of Al slab and alumina matrix are described by their bulk macroscopic dielectric functions ${\epsilon }_{Ge}(\omega )$, ${\epsilon }_{Al}(\omega )$ and ${\epsilon }_{M}(\omega )$. The Ge/Al nanoparticles form tetragonal superlattice with lateral and perpendicular lattice parameters *a* and *c*, respectively.

Modelling of Ge/Al_2_O_3_-matrix and Al-/Al_2_O_3_-matrix composite can be modeled as tetragonal supperlattice with lateral and perpendicular lattice parameter *a* and *c*, respectively, as illustrated in Fig. 2(a,b), while Ge/Al-shell/Al_2_O_3_-matrix composite has the same arrangement of nanoparticles but they consists of spherical Ge-core of radius *R*_*C*_ and Al-shell of outer radius *R*_*S*_ immersed in alumina matrix, as illustrated in Fig. 2(c). First we shell consider just one Ge-core/Al-shell nanoparticle immersed in alumina matrix, where response properties of each component Ge-core, Al-shell and Al_2_O_3_ matrix is described by their macroscopic dielectric functions *ϵ*_*Ge*_(*ω*), *ϵ*_*Al*_(*ω*) and *ϵ*_*M*_(*ω*), respectively. Suppose that such system is under the influence of external homogenous electrical field **E** = **E**_0_$\hat{z}$. The general solution of Poisson equation Δ*ϕ* = 0 for the i-th region, using the spherical coordinates, is then

$${\varphi }_{i}(r,\theta )=\mathop{\sum }\limits_{l\mathrm{=0}}^{\infty }\,\{{A}_{l}^{i}{r}^{l}+{B}_{l}^{i}{r}^{-(l+\mathrm{1)}}\}{P}_{l}(cos\theta );\,i=\mathrm{1,}\,\mathrm{2,}\,3$$

where *P*_*l*_ are Legendre functions, *l* is orbital quantum number and *i* = 1, 2 and 3 stay for Ge-core, Al-shell and alumina matrix regions, respectively. After using tangential and normal boundary conditions

$$\begin{array}{rcl}{\frac{1}{r}\frac{\partial {\varphi }_{i}}{\partial \theta }|}_{{R}_{i}} & = & {\frac{1}{r}\frac{\partial {\varphi }_{i+1}}{\partial \theta }|}_{{R}_{i}},\\ {\epsilon }_{i}(\omega ){\frac{\partial {\varphi }_{i}}{\partial r}|}_{{R}_{i}} & = & {\epsilon }_{i+1}(\omega ){\frac{\partial {\varphi }_{i+1}}{\partial r}|}_{{R}_{i}}\end{array}$$

and also asymptotic boundary condition

$$\varphi (r\to \infty )=-\,{E}_{0}r\,\cos \,\theta ,$$

we can obtain the potentials *ϕ*_*i*_ in different regions i = 1, 2, 3. Here *R*_1_ = *R*_*C*_ and *R*_2_ = *R*_*S*_. This solutions give good description of potential and charge density distributions in macroscopic dielectric, however considering the nanometer size of regions *i* = 1, 2 the solutions *ϕ*_1,2_ are probably very rough approximation. However the quantity which represents averaging over microscopic charge density distribution within the nanoparticle and which therefore has more macroscopic nature is induced dipolar potential far away from nanoparticle (*r* ≫ *R*_*S*_). This potential represents the first non vanishing term (*l* = 1) in *ϕ*_3_(**r**,*θ*), i.e.

6 $${\varphi }_{3}(r,\theta )\approx {B}_{1}^{3}{r}^{-2}{P}_{1}(cos\theta \mathrm{)}.$$

After comparison of (6) with expression for point dipole potential *ϕ*_*dip*_(*r*) = *p* cos*θ*/*r* it is obvious that the coefficient *B*_1_^3^ has meaning of nanoparticle induced dipole momentum, i.e. *p* = *B*_1_^3^. After solving the Poisson equation for spherical boundary conditions we get the induced dipole momentum of one Ge-core/Al-shell nanoparticle *p* = *α*(*ω*)*E*_0_, where nanoparticle polarizability is given by

7 $$\alpha (\omega )={R}_{S}^{3}\frac{[{\epsilon }_{Al}(\omega )-{\epsilon }_{M}(\omega )][{\epsilon }_{Ge}(\omega )+2{\epsilon }_{Al}(\omega )]-\mathrm{[2}{\epsilon }_{Al}(\omega )+{\epsilon }_{M}(\omega )][{\epsilon }_{Al}(\omega )-{\epsilon }_{Ge}(\omega )]{(\frac{{R}_{C}}{{R}_{S}})}^{3}}{[{\epsilon }_{Al}(\omega )+2{\epsilon }_{M}(\omega )][{\epsilon }_{Ge}(\omega )+2{\epsilon }_{Al}(\omega )]-\mathrm{2[}{\epsilon }_{M}(\omega )-{\epsilon }_{Al}(\omega )][{\epsilon }_{Ge}(\omega )-{\epsilon }_{Al}(\omega )]{(\frac{{R}_{C}}{{R}_{S}})}^{3}}\mathrm{}.$$

Moreover, considering that the system consists of lattice of polarizable Ge/Al-shell nanoparticles immersed in the polarizable alumina background, instead to bare external field **E**_0_ the system responds to screened macroscopic electrical field **E**. This means that the induced dipole momentum density is **P** = *χ*_*eff*_(*ω*)**E**, where effective susceptibility can be written as *χ*_*eff*_(*ω*) = [*ϵ*_*M*_(*ω*) − 1]/4*π* + *nα*(*ω*) and where *n* represents the Ge/Al-shell nanoparticle concentration. After using the connection between effective dielectric function and effective susceptibility *ϵ*_*eff*_ = 1 + 4*πχ*_*eff*_ the effective dielectric function of array of Ge/Al-shell nanoparticles in alumina matrix becomes

8 $${\epsilon }_{eff}^{(c)}(\omega )={\epsilon }_{M}(\omega )+4\pi n\alpha (\omega \mathrm{)}.$$

For larger metallic concentrations the metallic spheres increase and become to overlap forming undefined Al/alumina matrix boundaries. The limiting case showing Ge spheres placed in metallic slab of thickness *d*, is illustrated in Fig. 2(d), and the real one is a combination of the cases shown in the panels (c) and (d) of Fig.2, as illustrated in Fig. 2(e). For such auxiliary boundary conditions the Poisson equation cannot be solved analytically. However, in the lowest approximation, this situation can be modeled by Ge spheres placed in metallic slab of thickness *d*, as is illustrated in Fig. 2(d). For such model system the Poisson equation can be solved analytically and its effective dielectric function can be written as

9 $${\epsilon }_{eff}^{(d)}(\omega )={\epsilon }_{B}(\omega )+4\pi n{R}_{C}^{3}\frac{{\epsilon }_{Ge}(\omega )-{\epsilon }_{Al}(\omega )}{{\epsilon }_{Ge}(\omega )+2{\epsilon }_{Al}(\omega )},$$

where

$${\epsilon }_{B}(\omega )=\frac{d}{c}{\epsilon }_{Al}(\omega )+(1-\frac{d}{c}){\epsilon }_{M}(\omega )$$

represents the effective background dielectric function. Finally, if system in Fig. 2(e) is considered as some mixture of systems in Fig. 2(c,d) its effective dielectric function can be approximated as linear combination

10 $${\epsilon }_{eff}^{(e)}(\omega )=\alpha {\epsilon }_{eff}^{(c)}(\omega )+\mathrm{(1}-\alpha ){\epsilon }_{eff}^{(d)}(\omega );\,\alpha \in \mathrm{[0,}\,\mathrm{1]}.$$

Therefore, if *α* = 1 the system consists of purely Ge-core/Al-shell nanoparticles and if *α* = 0 the system consists of Ge nanospheres placed in Al slab.

## Results and Discussion

**Figure 3:**
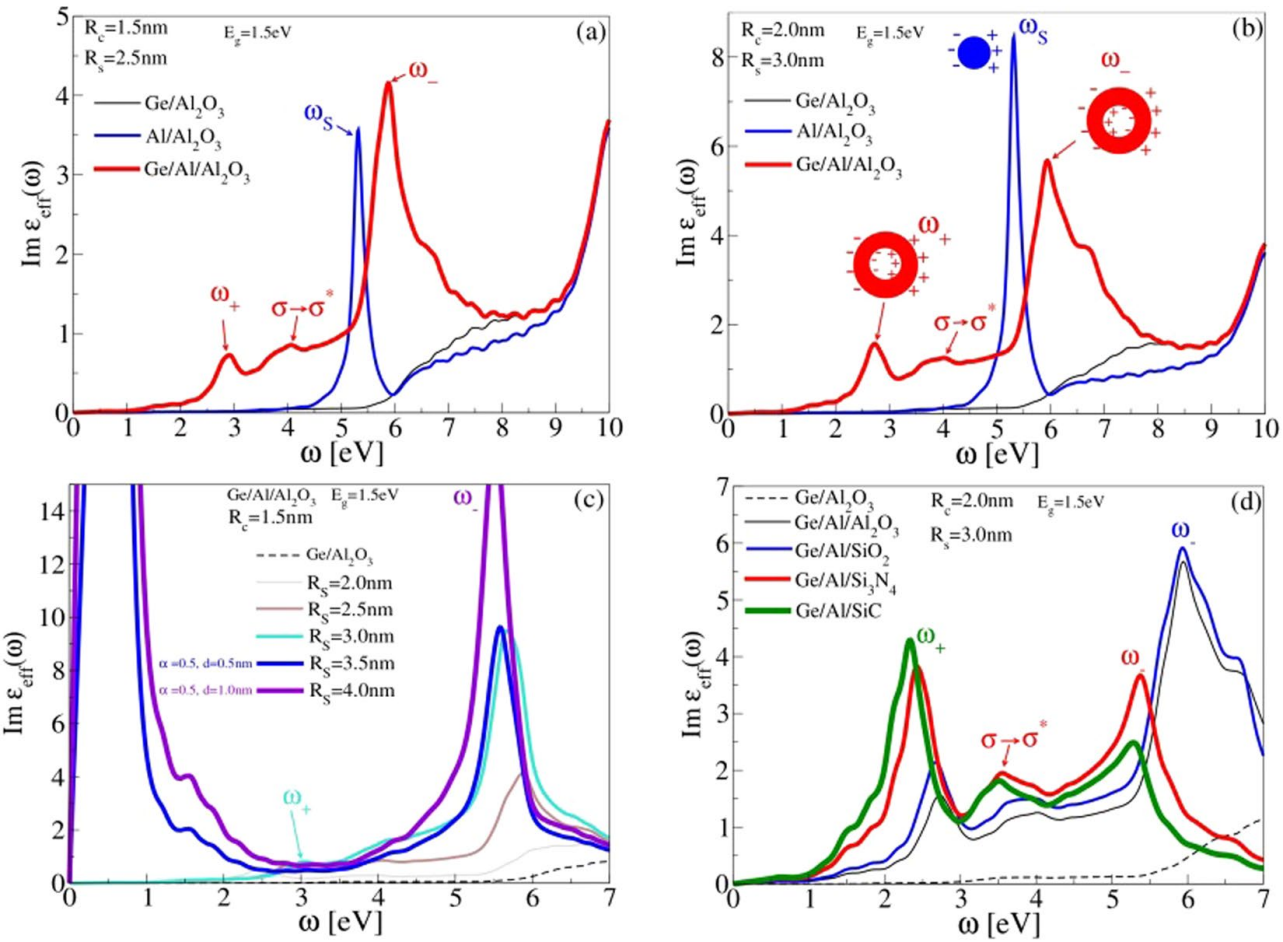
(**a**) Imaginary part of effective dielectric function of spherical Ge (thin black), Al (blue) and Ge/Al-shell (thick red) nanoparticles (also illustrated in Fig. 2(a–c), respectively) of inner radius *R*_*C*_ = 1.5 nm and outer radius *R*_*S*_ = 2.5 nm placed in Al_2_O_3_ matrix. (**b**) The same as for (**a**) except that inner radius is *R*_*C*_ = 2 nm and outer radius is *R*_*S*_ = 3.0 nm. (**c**) The imaginary part of effective dielectric function of Ge/Al-shell nanoparticles for various outer radii *R*_*S*_ = 2 nm(grey), 2.5 nm(brown), 3.0 nm(turquoise), 3.5 nm(blue) and 4.0 nm(violet). The inner Ge spherical core radius is *R*_*C*_ = 1.5 nm. For *R*_*S*_ = 3.5 and 4.0 the spherical shells become to overlap forming the nanostructure similar to one shown in Fig. 2(e) such that effective dielectric function is modeled by Eq.10 and parameters (*α* = 0.5, *d* = 0.5 nm) and (*α* = 0.5, *d* = 1 nm), respectively. Black dashed line shows the imaginary part of effective dielectric function of Ge-spheres of radius *R*_*C*_ = 1.5 nm in Al_2_O_3_ matrix, for comparison. (**d**) The imaginary part of effective dielectric function of Ge/Al-shell nanoparticles of inner radius *R*_*C*_ = 2 nm and outer radius *R*_*S*_ = 3.0 nm in various dielectric matrices Al_2_O_3_ (black), SiO_2_ (blue), Si_3_N_4_ (red) and SiC (green). Black dashed line shows the same as for (**c**) except that *R*_*C*_ = 2 nm.

The main aim of this research is to investigate the optical absorption of differently designed Ge/Al nanoparticle arrays immersed in various dielectric environments in order to propose the system with the most favorable optical properties in infra-red (IR) *ω* ~ 0–1.5 eV, visible (VIS) *ω* ~ 1.5–3.5 eV and ultraviolet *ω* ~ 3.5–10 eV frequency ranges. The optical absorption or electromagnetic energy dissipation rate can be easily connected with imaginary part of effective dilectric function (ℑ*ϵ*_*eff*_) explained in previous section. Figure 3(a) shows the imaginary part of effective dielectric function (Eqs.7 and 8) of array of spherical Ge (thin black), Al (blue) and Ge/Al-shell (thick red) nanoparticles, also sketched in Fig. 2(a–c), respectively. The nanoparticles are immersed in alumina (Al_2_O_3_) matrix and have inner radius is *R*_*C*_ = 1.5 nm and outer radius is *R*_*S*_ = 2.5 nm.

In frequency range *ω* < 6 *eV* the array of spherical Ge nanoparticle has low absorption and for larger frequencies the absorption gradually increases. It can be notice that the imaginary part of Al_2_O_3_ macroscopic dielectric function, shown in Fig. 1(c), has very similar trend, with optical absorption onset also at *ω* ≈ 6 eV. This suggests that in the system of spherical Ge nanoparticles the light will be dominantly absorbed by Al_2_O_3_ matrix and Ge nanoparticles do not play any important role.

However, if Ge-spheres are replaced by Al-spheres the situation changes significantly. Absorption of array of Al-spheres shows sharp peak at *ω*_*S*_ = 5.3 eV which corresponds to dipolar plasmon or Mie resonance which represents charge density fluctuations at the Al-sphere/Al_2_O_3_ interface. Charge density displacement corresponding to dipolar plasmon *ω*_*S*_ are sketched in Fig. 3(b). In the simple jellium model Al-sphere/Al_2_O_3_-matrix plasmon frequency can be estimated as ${\omega }_{S}={\omega }_{p}/\sqrt{1+2{\epsilon }_{M}}\approx 5.7$ eV (After solving the Poisson equation and implementing the spherical boundary conditions the effective dielectric function of array of Al-spheres in Al_2_O_3_ dielectric matrix becomes ${\epsilon }_{eff}(\omega \mathrm{)=1}+4\pi n{R}_{C}^{3}\frac{{\epsilon }_{Al}(\omega )-{\epsilon }_{M}(\omega )}{{\epsilon }_{Al}(\omega )+2{\epsilon }_{M}(\omega )}\mathrm{}.$ After using Drude approximation ${\epsilon }_{Al}(\omega \mathrm{)=1}-\frac{{\omega }_{p}^{2}}{{\omega }^{2}},$ and static approximation ${\omega }_{M}(\omega )\approx {\omega }_{M}(\omega =0)={\omega }_{M}$ the zero of denominator in ${\epsilon }_{eff}$ becomes ${\omega }_{S}={\omega }_{p}/\sqrt{1+2{\epsilon }_{M}}\mathrm{}.$), where we used the Al bulk plasmon frequency *ω*_*p*_ ≈ 15 *eV* and alumina static dielectric constant *ϵ*_*M*_ = 3 (see Fig. 1(c)). This nicely agrees with blue peak at *ω*_*S*_ ≈ 5.3 eV in Fig. 3(a). In the frequency interval *ω* &lt; *ω*_*S*_ the absorption of Al-spheres, similar to the case of Ge-spheres, becomes negligible. Therefore, even this system supports strong plasmon, it is very narrowly localized in UV frequency range and absorption in VIS frequency range remains zero. The absorption properties in VIS frequency range can be improved such that instead of using one-component spherical nanoparticles we can use two-component spherical nanoparticles which consists of semiconducting core and metallic shell (see Fig. 2(c)).

The thick red line in Fig. 3(a) shows that absorption spectra drastically change if Ge-spheres are encapsulated by Al-spherical shell. It can be seen that in absorption spectra dominate two broad peaks, intensive one in UV frequency range, at *ω*_−_ = 5.9 eV, and second weaker one at *ω*_+_ ≈ 2.9 eV. Second peak is responsible for substantial enhancement of absorption in VIS frequency range.

Similar to metallic sphere which supports dipolar plasmon *ω*_*S*_ the metallic spherical shell supports two dipolar plasmons, bonding *ω*_+_, and anti-bonding *ω*_−_, whose charge density displacements are sketched in Fig. 3(b). Two broad peaks in Fig. 3(a) are Landau damped by Ge and by Al_2_O_3_ *σ* → σ^*^ interband continuum. Plasmon *ω*_+_ is dominantly damped by low energy Ge and *ω*_−_ by high energy Al_2_O_3_ interband transitions. Therefore, this Ge/Al-shell configuration is quite favorable considering that it enables new plasmon *ω*_+_ which falls in VIS frequency range which, even weak and broad, substantially enhance absorption. However, as we shall see, the intensity and frequency of this peak can be additionally manipulated, e.g. by changing the size of nanoparticles, by changing the dielectric matrix or by changing the semiconducting core.

Figure 3(b) shows the same as Fig. 3(a) except that we change the size of nanoparticles such that the iner radius is now *R*_*C*_ = 2.0 nm and outer radius is *R*_*S*_ = 3.0 nm. As can be seen the array of Al-spheres (blue line) supports plasmon resonance *ω*_*S*_ which intensity is more than twice stronger and which frequency does not change in comparison with plasmon in Fig. 3(a). The latter is consistent with theory of Mie resonances in small spherical nanoparticles (retardation effects are neglected) when frequency of dipolar plasmon (*l* = 1) do not depend on sphere radius^30^.

Absorption in Ge/Al-shell configuration is shown by thick red line. In comparison with Fig. 3(a) the absorption of plasmon *ω*_−_ is more intensive and additionally widened. Absorption of plasmon *ω*_+_ is enhanced about twice. The peak *ω*_+_ is also slightly (about 0.2 eV) red shifted. Therefore, as expected, increase in nanoparticle size (larger Ge core the same Al shell thickness) enhances absorption in VIS frequency range. The appearance of third very broad peak at *ω* ≈ 4 eV in both Fig. 3(a,b) is very intriguing. Namely, it is not plasmon supported by metallic shell, but also it should not belong to interband transitions in germanium core, considering that absorption in germanium core (thin black line) is strongly suppressed in this frequency range. However, is should be taken into account that when *ω*_±_ plasmons absorb light they create strong evanescent electrical field which could enable enhanced absorption to interband *σ* → σ^*^ transition in germanium core. This phenomenon is very similar to enhanced near field sensing or enhanced Raman spectroscopy techniques. There the localized plasmon near field is exploited to excite electronic or vibrational modes in individual molecules or in biological or chemical compounds^10,31–34^. Therefore, the broad peak very likely represents the absorption to germanium core interband transitions *σ* → σ^*^ which are strongly enhanced (about 20 times) by *ω*_±_ plasmons. A detailed analysis of the nature of the observed plasmon modes is undergoing and will be topic of a future work.

In order to explore how the absorption in array of Ge/Al-shell nanoparticles immersed in Al_2_O_3_ matrix depends on Al-shell thickness the Fig. 3(c) shows the imaginary part of effective dielectric function for various Al-shells of outer radii *R*_*S*_ = 2 nm(grey), 2.5 nm(brown), 3.0 nm(turquoise), 3.5 nm(blue) and 4.0 nm(violet). For the inner, Ge spherical core, radius we take *R*_*C*_ = 1.5 nm. Also, for *R*_*S*_ = 3.5 and 4.0 nm the spherical shells become to overlap forming the nanostructure similar to one shown in Fig. 2(e) such that effective dielectric function is modeled by Eq. 10 where we use parameters (*α* = 0.5, *d* = 0.5 nm) and (*α* = 0.5, *d* = 1 nm), respectively. Black dashed line shows the imaginary part of effective dielectric function of Ge-spheres in Al_2_O_3_ matrix of radius *R*_*C*_ = 1.5 nm, for comparison. This graphs demonstrate very fast increase of absorption to plasmon *ω*_−_ with Al-shell thickness. On the other hand absorption to plasmon *ω*_+_ very slowly increases and remains very small. The intensity increase is followed by small red shift of plasmon *ω*_−_ and blue shift of plasmon *ω*_+_. For larger thicknesses *R*_*S*_ = 3.5 and 4.0 nm the Al shells become to overlap, the system becomes conductive and, besides the plasmon peak, strong Drude peak in IR frequency range appears. Obviously this system support strong plasmon resonances *ω*_−_ which lies in UV frequency range and which is therefore, not very promising for chemical sensing or optoelectronic applications. For this applications it would be more preferable if the system would supports wide plasmon resonance covering the IR and/or VIS frequency range. One possibility to improve this property is to shift plasmon *ω*_−_ to lower frequencies or to increase intensity of *ω*_+_ plasmon, which already lies in VIS frequency range. This could be achieved e.g. by using another dielectric matrix in which Ge/Al-shell particles would be immersed. Figure 3(d) shows the absorption of array of Ge/Al-shell nanoparticles of inner radius *R*_*C*_ = 2 nm and outer radius *R*_*S*_ = 3.0 nm in various dielectric matrices Al_2_O_3_ (black), SiO_2_ (blue), Si_3_N_4_ (red) and SiC (green). Black dashed line shows the imaginary part of effective dielectric function of Ge-spheres of radius *R*_*C*_ = 2 nm in Al_2_O_3_ matrix, for comparison. Due to their relatively large band gap the dielectric functions of SiO_2_, Si_3_N_4_ and SiC are approximated by their static values ${\epsilon }_{Si{O}_{2}}$ ≈ 3.9, ${\epsilon }_{S{i}_{3}{N}_{4}}$ ≈ 7.5 and *ϵ*_*SiC*_ ≈ 9.6, respectively. We have proven that this approximation gives satisfactory good results for Al_2_O_3_ matrix, when we have replaced the macroscopic dielectric function, shown in Fig. 1(c), by its statical value ${\epsilon }_{A{l}_{2}{O}_{3}}\approx 3.0$. For the case of SiO_2_ which has dielectric constant similar to Al_2_O_3_ the plasmon resonances *ω*_±_ almost does not change, although the absorption in VIS frequency interval slightly increases, in comparison with Al_2_O_3_. For the case of Si_3_N_4_ and SiC (which have two or three times larger dielectric constants then Al_2_O_3_) the intensity of plasmon resonance *ω*_−_ substantially decreases and becomes red shifted for about 0.5 eV, such that it still remains in UV frequency interval. In the same time broad plasmon resonances *ω*_+_ become red shifted by about 0.4 eV such that now they fall exactly in the middle of VIS frequency range (${\omega }_{+} \sim 2.5$ eV). Moreover, what is especially important, now the intensity of *ω*_+_ plasmons become about three times larger than in the case when nanoparticles are immersed in Al_2_O_3_. This suggests that materials which consists of Ge/Al-shell nanoparticles immersed in wide band-gap and larger dielectric constant (*ϵ* > 5) dielectric matrix will be good absorbers of the most intensive sunlight frequencies and therefore suitable in solar-cells applications. It may also be noted that larger dielectric constant causes red shift of interband *σ* → σ^*^ transitions which also become more prominent peak.

Here we should mention that above analisis do not taken into account the ‘dielectric mismatch’ effects and the only way how we include the quantum confinement effect, which is also very sensitive on dielectric matrix used, is through shift in band-gap Δ. However, some recent studies^35–37^ show that the ‘dielectric mismatch’ effects can substantially modify the optical spectrum of CdS quantum dot. This studies represent an more extensive and sophysticate approach which consider the electron energy levels in quantum-dot/dielectric herostructure using multibands effective-mass Hamiltonian and envelope-wave function approach. In this model the electron and holes are described by effective mass parabolic bands whose energies at Γ-point also called confinement energies depends on quantum dot size and shape. The envelope wave functions (and therefore the confinement energies) are dermined by boundary conditions at quantum dot edges. The confinement energies are also very sensitive on ‘dielectric mismatch’ which defines the height and dispersivity of the potential barrier at quantum-dot/dielectric interface which then defines the dielectric confinement contribution to confinement energy. This means that in our case confinement energy will strongly depend on dielectric matrix used and therefore influence the nanoparticle electronic structure. Consequently, this could affect the exciton energies and absorption spectra and threfore the results shown in Fig. 3(d). Here, the ‘dielectric mismatch’ effects could eventually be include such that for different dielectric matrices we simultaneously change the band gap Δ. However, that approach would also be very questionable because the germanium core is in contact with dielectric matrix through Al-shell which additionally complicate the situation. However we should emphasize here that this investigatinin is not aimed to explore the single particle electron-hole transitions and excitons in Ge-core/Al-shell/Al_2_O_3_-matrix hetersostructure but rather to explore the modiffication of strong plasmon resonances in Al-shell. Such that the point of Fig. 3(d) is to demonstrate how the Ge-core and Al_2_O_3_-matrix screening affect the *ω*_+_ plasmon intensity. But, of course, we should be aware that the ‘dielectric mismatch’ effects can compromise our conclusions about other electronic excitations originating from Ge-core.

**Figure 4:**
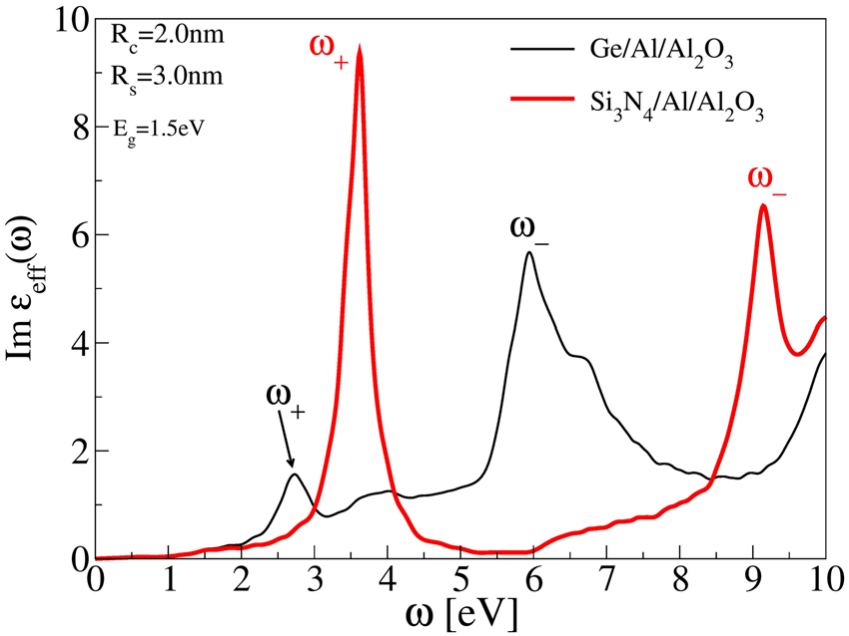
Imaginary part of effective dielectric function in array of (thin black) Ge/Al-shell and (thick red) Si_3_N_4_/Al-shell nanoparticles of inner radius *R*_*C*_ = 2 nm and outer radius *R*_*S*_ = 3.0 nm placed in Al_2_O_3_ matrix.

Another possibility to enhance optical absorption in VIS frequency range is to replace the Ge-core with other, large band-gap semiconductors which would reduce *ω*_+_ plasmon losses to *σ* → σ^*^ interband electron-hole excitations in semiconducting core. In order to demonstrate this effect, we replace the Ge-core by Si_3_N_4_-core which has desired band gap of Δ ≈ 5 *eV*. Thin black line in Fig. 4 shows the imaginary part of effective dielectric function in array of Ge/Al-shell nanoparticles of inner radius *R*_*C*_ = 2 nm and outer radius *R*_*S*_ = 3.0 nm immersed in Al_2_O_3_ matrix. For comparison, the thick red line shows the same except that Ge-core is replaced by Si_3_N_4_-core. It can be notice that Si_3_N_4_-core causes strong enhancement (about 6 times) or recuperation of *ω*_+_ plasmon which, even blue shifted, still remains in VIS frequency region. In the same time *ω*_−_ plasmon is blue shifted in far-UV frequency region.

**Figure 5:**
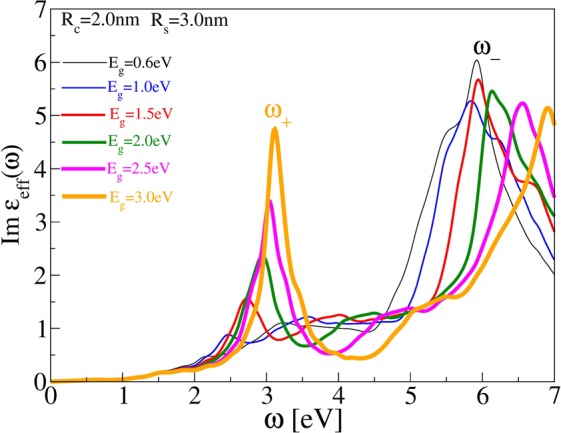
Imaginary part of effective dielectric function in array of Ge/Al-shell nanoparticles of inner radius *R*_*C*_ = 2 nm and outer radius *R*_*S*_ = 3.0 nm placed in Al_2_O_3_ matrix. The Ge band-gap increases due to quantum size effects as Δ = 0.66 eV(black), Δ = 1.0 eV(blue), Δ = 1.5 eV(red), Δ = 2.0 eV(green), Δ = 2.5 eV(magenta) and Δ = 3.0 eV(orange). The line thickness increases with band gap.

Now we shall demonstrate that the plasmon *ω*_+_ appears in Ge/Al-shell nanoparticles solely due to quantum-size effects. Namely, the nanometer confinement can significantly increase semiconductor band-gap and thus reduces *ω*_+_ plasmon losses to interband *σ* → σ^*^ transitions. In order to simulate the development of *ω*_+_ plasmon due to quantum-size effect we increase the germanium bulk band-gap from Δ = 0.66 eV to several larger values. Figure 5 shows imaginary part of effective dielectric function in array of Ge/Al-shell nanoparticles of inner radius *R*_*C*_ = 2 nm and outer radius *R*_*S*_ = 3.0 nm placed in Al_2_O_3_ matrix for various Ge band-gaps Δ = 0.66 eV (black), Δ = 1.0 eV(blue), Δ = 1.5 eV(red), Δ = 2.0 eV(green), Δ = 2.5 eV(magenta) and Δ = 3.0 eV(orange). This graphs nicely demonstrate that for bulk Ge band-gap (Δ = 0.66 eV) the plasmon *ω*_+_ almost does not exist, however, its intensity rapidly increases how band gap increases. Also, increasing band-gap causes blue shift of *ω*_+_ plasmon, but such that its spectral weight is still mostly localized in VIS frequency range. For example, for the largest band-gap (Δ = 3.0 eV) this plasmon becomes very intensive peak localized in violet frequency range ${\omega }_{+} \sim 3.1$ eV. The plasmon *ω*_−_ also becomes blue shifted and its intensity slowly decreases how band-gap increases.

### Comparison with experiment

In order to explore the possible application of proposed plasmonic resonances in optoelectronic devices some conclusions made will bring into connect with recent experimental realization of Ge-core Al-shell nanoparticles in alumina (L. B., V. D., *et al*., *Ge-metal core-shell nanoparticles in alumina matrix: structure, chemical bonds and absorption*, and their optical properties. The proposed simulations can explain the experiment just qualitatively considering that experimentally fabricated nanoparticles are not perfect concentric spheres, but rather non-concentric ellipsoids and also the samples are not perfectly clean, etc.

**Figure 6:**
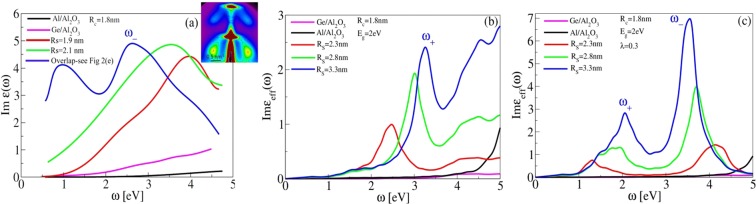
(**a**) Ellipsometry measurements of the imaginary part of effective dielectric function in array of Ge nanoparticles (magenta), Al nanoparticles (black) and in array of Ge/Al-shell nanoparticles where red, green and blue lines show results of measurements for thin, medium thick and thick Al-shell, respectively^18^. The average germanium core radius is *R*_*S*_ ≈ 1.8 nm. (**b**) The theoretical simulation of imaginary part of effective dielectric function in array of Ge nanospheres (magenta), Al nanospheres (black) and Ge/Al-shell nanoparticles, where inner (core) radius is *R*_*C*_ = 1.8 nm and outer radii are *R*_*S*_ = 2.3 nm (red), *R*_*S*_ = 2.8 nm (green) and *R*_*S*_ = 3.3 nm (blue). (**c**) The same as in (**b**) except that Al-shell dielectric function is simulated by Eq. 11, where *α* = 0.3. Nanoparticles are immersed in Al_2_O_3_ matrix. The germanium band-gap is here, due to quantum confinement, taken to be Δ = 2 eV.

Figure 6(a) shows the imaginary part of effective dielectric function determined from ellipsometry measurements in array of different nanoparticles immersed in Al_2_O_3_ matrix^18^. The average germanium core radius is *R*_*C*_ ≈ 1.8 nm. It can be seen that absorption in Ge nanoparticles (magenta) and in Al nanoparticles (black) is much weaker than absorption in Ge/Al-shell nanoparticles, whose shell thickness appears as thin (red), medium thick (green) and thick (blue), which anticipates (already described) advantages of using metallic-shell nanostructures. It can be seen that absorption in array of Ge/Al-shell nanoparticles shows peak which frequency decreases and intensity slowly increases how shell thickness increases. Also in the case of thick shell (blue) the second peak appears in the absorption spectra. We shall try to explain this experiment using the above explored model.

Figure 6(b) shows the theoretical simulation of imaginary part of effective dielectric function in array of Ge nanospheres (magenta), Al nanospheres (black) and Ge/Al-shell nanoparticles where the inner (core) radius is *R*_*C*_ = 1.8 nm and outer radius increases as *R*_*S*_ = 2.3 nm (red), *R*_*S*_ = 2.8 nm (green) and *R*_*S*_ = 3.3 nm (blue). The germanium band-gap is here, due to quantum confinement, taken to be Δ = 2 eV. As can be seen, in the shown frequency interval the absorption in array of Ge/Al-shell nanoparticles is much stronger than in array of Ge or Al nanospheres. Also it can be seen that metallic-shell spectrum shows peak which intensity increases with shell thickness, however, its frequency also increases with thickness which is contrary to the experiment. These peaks correspond to plasmons *ω*_+_ whose frequency, as theoretically expected, increases with shell thickness. In the same time the frequency of plasmon *ω*_−_ (not seen here because its frequency is *ω*_−_ > 5 eV) decreases with shell thickness and finally for very large thicknesses (*R*_*S*_ ≫ *R*_*C*_) degenerate with plasmon *ω*_+_. This theoretical scenario suggests that the decreasing antibonding plasmon *ω*_−_ should be the one which appears in experimental spectra and the second peak which appears for thick shell (blue line in Fig. 6(a)) maybe corresponds to plasmon *ω*_+_. However, the simulated frequencies *ω*_±_ are much larger than experimental. One possibility why the experimental frequencies *ω*_±_ are much smaller than simulated is because the Al- shells fully or partially overlap for that film (see the model in Fig. 2(e)). The simulations for that case of material structure are shown in Fig. 3(c), where an intensive peak is visible at low energies. Another possibility why the experimental frequencies *ω*_±_ are much smaller than simulated is the fact that fabricated metallic shells are not fully clean, i.e. it is possible that they contain some admixture of alumina which would lower the plasmon frequency. If this is the case, then the spherical shell can be modeled as two components linear dielectric which polarizability is *χ*_*shell*_ = *λχ*_*Al*_ + (1−*λ*)${\chi }_{A{l}_{2}{O}_{3}}$; *λ* ∈ [0, 1], which after using relation *ϵ*_*i*_ = 1 + 4*πχ*_*i*_; *i* = Al, Al_2_O_3_, leads modified metallic-shell dielectric function

11 $${\epsilon }_{shell}(\omega )=\lambda \,{\epsilon }_{Al}(\omega )+\mathrm{(1}-\lambda )\,{\epsilon }_{A{l}_{2}{O}_{3}}(\omega \mathrm{)}.$$

Figure 6(c) shows the same as Fig. 6(b) except that the Al-shell dielectric function is replaced by modified metallic-shell dielectric function (11), where *λ* = 0.3. As can be seen the plasmon frequencies *ω*_±_ are now much lower and comparable with experimental values. Intensity of *ω*_−_ plasmon increases and its frequency decreases with shell thickness and in the same time another, weaker plasmon *ω*_+_ is present which is all consistent with experimental trend. This scenario obviously suggests large contamination of Al-shell by Al_2_O_3_. Contamination of Al-shell by Al_2_O_3_ is realistic because Al bonds all oxygen to itself, so Ge stays oxygen free^18^. Another fact that exist in the experiment and it is not taken into account in the simulation, is distribution of the nanoparticle sizes. It affects both, core and shell. According to the experiment, the standard deviation of the size distributions is 1.5–2.2  nm for smallest and largest shell size, respectively^18^. Obviously, the size distribution broadens the peaks shown in the simulations. Finally, electron confinement in thin Al shells may result in intrinsic size effects that reduce electron damping and broaden the resonances^38^.Therefore, all this should be taken into account for the realistic comparison of the experimental and theoretical data.

## Conclusions

We provided the theoretical simulation of optical absorption in lattice of various spherical Al, Ge, Ge-core/Al-shell nanoparticles immersed in Al_2_O_3_ matrix. It is demonstrated that the absorption is strongly enhanced when Ge spheres are encapsulated by Al-shell. The lattice of Ge-core/Al-shell nanoparticles is recently experimentally fabricated and explored using various different techniques^18^. Strong enhancement of the absorption with respect to only Ge nanoparticles is observed there, and the measured spectra agrees well with the simulations presented here. The Ge-core/Al-shell lattice posses two absorption peaks in analogy to bonding (*ω*_+_) and anti-bonding (*ω*_−_) plasmons appearing in metal-dielectric core-shell particles which lie in VIS and UV frequency range, respectively. The absorption to *ω*_+_ plasmon, in VIS frequency range, enhances additional 3 times when Al_2_O_3_ is replaced by larger dielectric constant insulator, such as SiC. Replacement of Ge-core by wide band-gap insulator, such as Si_3_N_4_, prevents plasmon decay to interband *σ* → σ^*^ electron-hole excitations in semiconducting core, and optical absorption to *ω*_+_ plasmon enhances additional 6 times. It is shown that in Ge-core/Al-shell system the *ω*_+_ plasmon exists only because of quantum-size effect (it causes widening of Ge band-gap) which prevents its decay to low-lying interband *σ* → σ^*^ electron-hole excitations in Ge-core. Strong optical absorption in VIS frequency range suggests that several nanometers large semiconducting-core/metallic-shell nanoparticles could be very suitable for optoelectronic applications. For example, it can improve absorption of visible light in photovoltaic devices or enhance emission in light emitting devices. Also, it can serve as highly light absorbing platform in biological or chemical sensing.
